# “I think I could have coped if I was sleeping better”: Sleep across the trajectory of caring for a family member with dementia

**DOI:** 10.1177/14713012231166744

**Published:** 2023-03-30

**Authors:** Rosemary Gibson, Amy Helm, Isabelle Ross, Philippa Gander, Mary Breheny

**Affiliations:** School of Psychology, 6420 Massey University, Palmerston North, New Zealand; Sleep/Wake Research Centre, School of Health Sciences, 6420 Massey University, Wellington, New Zealand; Department of Sleep/Wake Research Centre, School of Health Sciences, 6420 Massey University, Wellington, New Zealand; School of Health, 8491 Victoria University of Wellington, Wellington, New Zealand

**Keywords:** carers, dementia, grief, sleep, themes, transitions

## Abstract

Dementia-related sleep changes can lead to disruptions among families living with dementia which can jeopardise carers’ wellbeing and ability to provide support. This research explores and represents the sleep of family caregivers across the trajectory of caring, before, during, and after the key period of their care recipient moving into residential care. The focus of this paper is viewing dementia caregiving as a trajectory, characterised by care needs which change over time. Semi-structured interviews were conducted with 20 carers whose family member with dementia had transitioned into residential care within the prior 2 years. Themes constructed from these interviews indicated that sleep was linked to earlier life course patterns as well as to significant moments of transition in the caregiving journey. As dementia progressed, carers’ sleep progressively worsened in relation to the less predictable nature of dementia-symptoms, difficulty maintaining routines, and constant responsibilities creating a state of high alert. Carers attempted to facilitate better sleep and wellbeing for their family member, often sacrificing their own self-care. Around the care transition period, some cares reported not realising how sleep deprived they were; for others the busy momentum continued. After the transition, many carers acknowledged that they were exhausted, although many had not realised this while providing home-based care. Post-transition, many carers reported ongoing sleep disruptions associated with poor sleep habits established whilst caring, insomnia or nightmares and grief. Carers were optimistic that their sleep would improve with time and many were enjoying sleeping according to their own preferences. The sleep experience of family carers is unique and includes tensions between their essential need for sleep and the experience of care as self-sacrifice. Findings have implications for timely support and interventions for families living with dementia.

## Introduction

Sleep is increasingly recognised as a pillar of physical and mental health, as well as facilitating ageing well (Foley et al., 2004; Gibson, Gander, Kepa, Moyes, & Kerse, 2020). Sleep disturbances are common among people with dementia, with symptoms commonly including irregular sleep timing, increased symptoms of insomnia, night-time agitation or confused behaviours, nightmares, and daytime sleepiness (Bliwise, 2004; Gibson & Gander, 2020; Gibson et al., 2014; McCurry & Ancoli-Israel, 2003). Many informal carers have sleep disturbances secondary to the care recipient’s sleep or in relation to the full-time nature of caregiving and stress related to the situation (Gibson & Gander, 2020; Maun et al., 2018; McCurry et al., 2015; Reynolds et al., 2020).

Using the Insomnia Severity Index (Morin et al., 2011), a New Zealand (NZ) postal survey found 23% of people actively supporting a family member with cognitive impairment or dementia indicated had a moderate to severe sleep problem and 42% a mild problem (Gibson & Gander, 2020). Having a moderate-severe sleep problem was independently associated with carers also rating their health and standards of living more poorly compared to those without sleep problems or with mild sleep problems. They also had a 74% increased odds of considering residential aged care (RAC) services for their family member for a move either imminently or within the year (Gibson & Gander, 2020). These findings highlighted the importance of considering carers’ sleep, alongside other factors, as an important factor for families affected by dementia to remain living well together at home.

Sleep health for dementia carers is complex and changeable. To understand this complexity, open-ended survey comments on experiences of sleep have been analysed. Unique themes concerning carers’ sleep were represented across Buysse’s (2014) five dimensions of sleep health (sleep duration, efficiency, timing, alertness, and satisfaction). Sleep is balanced amongst other responsibilities, time constraints, and living conditions (Gibson, Helm, Breheny, & Gander, 2020). Carers often described how their sleep disruptions changed across time, suggesting that the timing of support to assess and potentially address sleep problems is important.

Perceptions and expectations around sleep needs, disruptions and behaviour change with ageing as well as with transitions of waking life, responsibilities, and goals (Venn et al., 2013). Furthermore, sleep deprivation has, in itself, been identified as a barrier to recognising the impact of sleep loss on waking function and performance (Boardman et al., 2018). This has implications for waking safety and help-seeking but seldom explored from the perspective of dementia care.

The majority of research concerning sleep and dementia care has used cross-sectional surveys, actigraphy recordings, or interviews during the period when families are in the midst of managing dementia at home (Gao et al., 2019; McCurry et al., 2007). Studies concerning the sleep of residents of RAC facilities are also common (Alessi & Schnelle, 2000; Webster et al., 2020). Psychosocial life transitions are increasingly recognised as important factors affecting sleep. For example, periods such as retirement, moving home, trauma, or grief have all been identified as influencing sleep timing, increasing symptoms of insomnia (i.e. getting to sleep and staying asleep) or dreaming (Lavie, 2001; Lerdal et al., 2013; Myllyntausta et al., 2017). However, studies considering sleep around transitions within dementia care are underrepresented. Key transitions of interest here include aiding decision making around care needs, assisting the movement of a family member into RAC, life after the transition, and bereavement (Ashbourne et al., 2021; Sury et al., 2013). While these studies indicated some quantitative sleep changes among carers during such transitions, the nuanced situation and context of sleep requires qualitative representation.

The current research sought to better understand and represent the changing sleep experience with transitions relevant to dementia care. In particular, this research aimed to understand the effect of sleep problems on managing the caregiving situation, how sleep changes when a family member with dementia moves into RAC and explore carers’ sleep health and wellbeing thereafter. Throughout it is acknowledged that the caregiving role does not necessarily end with such transitions, but rather changes alongside them.

## Methods

### Participants and Data Collection

Twenty participants were recruited from a mailing list of people who had completed a previous postal survey concerning sleep and dementia-related care in NZ (Gibson & Gander, 2020). The original survey content and design was informed and piloted by Dementia Wellington staff and service users before distribution to the mailing lists of all 21 regional services associated with either Alzheimer’s NZ or Dementia NZ services (approximately 4720 surveys were distributed and 11% returned, see Gibson & Gander, 2020, for further details). Interview invitations were sent to 93 potential participants who lived either within the lower North Island of New Zealand or rurally. This recruitment strategy was designed to represent experiences of both urban and rural contexts. In order to capture the experiences and perspectives across the dementia and caregiving trajectory, participants were eligible if the person they supported had transitioned into RAC since they had contributed to the postal survey approximately 2 years earlier.

Participants completed a written consent form alongside a brief descriptive questionnaire by post (including demographic details of themselves and the care recipient, relationship, time spent caring, and time since movement to RAC). Current sleep quality was measured using the Pittsburgh Sleep Quality Index (PSQI). This provides a global score derived from items representing self-reported sleep quality, latency, duration, efficiency, disturbances, use of medications and daytime dysfunction. Scores range from 0 to 21 with “poor sleepers” defined as those scoring ≥5 (Buysse et al., 1989). Health status was measured using the comorbidity questionnaire to indicate diagnosed health conditions (Sangha et al., 2003).

Interviews were semi-structured and explored: the experience of providing informal care for a family member with dementia; how sleep changed for both parties as the disease progressed; how sleep problems were (or were not) managed; the role of sleep disturbances in the decisions around formal care needs; sleep around the time of the family member moving into RAC; and how family carers were sleeping after this transition (and, if applicable, death of the person with dementia). Interviews were conducted in the form of an informal conversation. Therefore, these topics were not approached in a structured form. Instead prompts were used if aspects had not been covered naturally during the interview. Thirteen interviews took place in participant’s homes in urban centres of the lower North Island. The remaining seven were conducted by telephone (with carers living in rural locations). The research was conducted in accordance with the requirements of the Central Health and Disability Ethics Committee guidelines (16/CEN/101/AM01).

### Data Analysis

Interview recordings ranged from 28 to 103 min (median 55 min) and were transcribed verbatim. Names of participants, family members, as well as key place names and care providers were excluded or changed to ensure anonymity. Following transcription, a thematic analysis was conducted to examine and represent the complexities of the participants’ sleep-related experiences within social, interpersonal and transitional contexts. This approach considers the participants accounts as representations of their own experiences and perceptions of sleep in the context of informal care and beyond (Attride-Stirling, 2001; Braun & Clarke, 2019). The transcripts were checked and corrected before coding using NVivo12 software. Initial coding of the interviews was inductive and focused on identifying recurrent patterns in the experience of sleep during dementia care. Codes were collated and organised into themes which were cross-checked against experiences for individual participants across time. This process identified sleep for caregivers as situated in terms of key transition points. Themes were then workshopped with the wider team to focus on how sleep changed across the caregiving trajectory. Five time points were identified as mentioned repeatedly across the interviews and as anchoring the accounts of sleep provided. These five points were sleep prior to care, sleep during care (while actively caring at home), sleep disruptions around the time of their family member transitioning to RAC (i.e., during the weeks immediately prior to or post their family member moving into care), sleep post transition to RAC, and, for those whose care recipient had since died, sleep following the death. Experiences of sleep were organised across these time points and illustrative quotes were selected to exemplify these experiences. Themes constructed from the analysis are presented in italics and illustrated with quotes (e.g., *“On high alert day and night”*), and are discussed and organised across the caregiving trajectory. Stories people told about their realisation of formal care needs and decision making were outside of the original research question regarding sleep changes. These are summarised elsewhere (Wright, 2020).

## Findings

**Figure 1.**
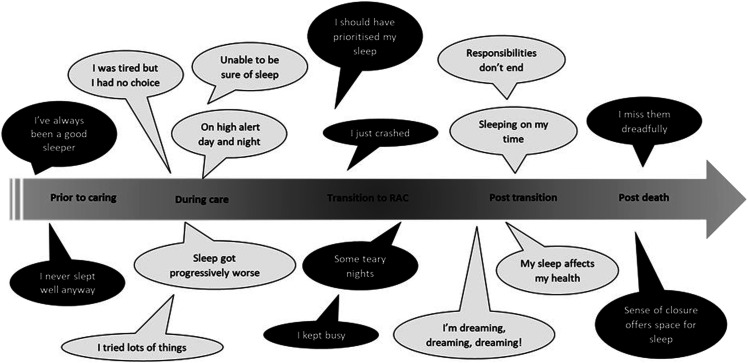
Themes of carers sleep experiences across key time points of dementia care.

The median age of participants was 75.5 years (range 24–87), 14 were female, and the majority (18) identified as NZ European ethnicity (one identified as Māori, the indigenous people of NZ, and one as Samoan). Seventeen had been an informal carer for their spouse. The other care recipients were a long-term cohabiting friend, a parent, and a grandparent. Eight of the care recipients had died since moving into RAC. At the time of the interview, participants mean PSQI score was 8.7 (SD 3.8) and 17 (85%) scored above the PSQI threshold (score of 5) denoting “poor sleep”. Carers had a median of two health conditions (range 0–5), with the most common being heart disease or high blood pressure (see Table 1). Themes are presented in Figure 1 organised by time points. These themes are described in more detail below.

**Table 1.** Description of participants.

Pseudonyms Carer (CR)	Interview mins.	Age	Care years	Months since RAC	Dementia	CR death	Carer PSQI
Sonia (and martin)	32	68	6.0	11.0	Mixed		7.0
Steven (and Karen)	55	83	3.0	10.5	AD		3.0
Sue (and Brent)	55	79	4.0	Missing	Prob. AD	Yes	13.0
Joe (and Fiona)	58	71	4.5	15.0	FTD		15.0
Sally (and Roy)	77	77	4.0	3.0	Unknown		7.0
Sarah (and George)	47	82	3.5	12.0	Vascular		10.7
Nathalie (and William)	47	64	3.5	16.0	Vascular		8.0
Pamela (and Tony)	64	75	5.0	8.0	Vascular		13.0
Grace (and Edward)	28	69	3.5	15.0	Vascular	Yes	12.0
Ruby (and Adam)	92	86	Missing	24.0	Unknown	Yes	5.0
Olivia (and Holly)^1^	40	73	3.5	13.0	AD		13.0
Eva (and Brenda)	35	24	1.5	5.3	AD		8.0
John (and Linda)	69	83	3.7	5.0	Vascular	Yes	8.0
Emma (and Matthew)^2^	61	66	3.0	Missing	FTD	Yes	8.5
Jacob (and Ivy)^2^	65	87	3.0	11.0	Unknown	Yes	4.2
Daniel (and Lucy)^2^	53	73	5.0	7.0	AD		15.0
Hannah (and Charles)^2^	35	76	8.0	12.0	AD		3.0
Harry (and Molly)^2^	47	81	2.7	10.0	Unknown	Yes	5.0
Nicole (and Derek)^2^	58	77	4.0	15.0	FTD	Yes	5.0
Moana (and grandfather)^2^	103	48	3.7	13.5	Vascular		10.0

CR = Care recipient. 1 = telephone interview by preference; 2 = rural dwelling (telephone interview) AD = Alzheimer’s Disease; FTD = Frontal Temporal dementia. PSQI = Pittsburgh Sleep Quality Index.

### Sleep Prior to Caring

Most participants began the interview by discussing their sleep experiences before caring, identifying their sleep on a continuum from poor to good. For example, the theme *“Never slept well anyway”* encompasses those who had experienced and overcome periods of poor sleep in the past:

…it’s the same. I've been a very bad sleeper anyway….and that’s no problem to me. Cos I'm never tired during the day. (Sonia, 68).

The theme *“Always been a good sleeper”* represents those who reported satisfactory sleep or feeling that they managed on little sleep anyway (e.g., as a shift worker etc.):

…with shift work, years ago. I was really good on night shifts…You know like, people couldn’t believe that I was so, amiable on nightshift if I was woken up or anything. Because it really didn’t bother me. (Nathalie, 64).

This existing sleep identity formed a basis for comparison which carers evaluated changes associated with care, as well as the strategies they already had in place to self-manage sleep. Sleep was represented as part of an established pattern of behaviours (sleeping well or badly) or as an established identity (a good or poor sleeper). Sleep whilst actively caring was considered in light of these patterns and identities.

### Sleep during Care

During the time of actively supporting their family member with symptoms of dementia, many of the carers described sleep disruptions related to the high responsibilities of care, i.e., physically and mentally supporting their family member in the night. The theme *“On high alert day and night”* reflected this sense of continuous responsibility:

you're always, even though you don’t realise it, slightly on edge and waiting for the next unexpected thing to happen. And that is very unsettling when it comes to sleeping. Because things do happen in the night. (Sarah, 82).

The theme *“Being unsure of sleep”* represents the unpredictable nature of care provision, particularly overnight. Care requirements overnight varied but common responsibilities included toileting or continence-related support, guiding the care recipient back to bed, and comforting them if they were unable to sleep or after nightmares and hallucinations. Many carers reported symptoms of insomnia. The ability to successfully get to sleep and stay asleep was limited due to the timing of sleep being dictated by that of the care recipient rather than their own preferences. Expecting to be woken or needed in the night limited their ability to sleep deeply. Feelings of stress or grief were also common among carers and affected the ability to sleep easily or well:

He’d go to bed early and then for – he’d want to go to bed at half past eight – ‘come on, come on to bed now’. And I said, ‘no I’ve – there’s a television programme I want to see’. ‘Well come to bed now’. I said, ‘you can go to sleep without me’. ‘No I can’t’. I said ‘you slept two hours this morning and two hours this afternoon without me’. I said, ‘you can go to sleep again tonight surely’. ‘No I can’t, I want you in here with me’. And I said, ‘well I’m not coming until this programme’s finished, so I can stop bellowing at me’. But it would start up again. And in the end, I’ve had to switch off the TV and go in …I’d go to bed…He’d be in bed a good hour before I was and he’d nag, nag, nag until I’d go in there too. And then he’d have a disturbed night and wake me up. (Ruby, 86).

A key theme regarding the active period of caring was that *“Sleep got progressively worse”.* This encompasses the gradual deterioration of carers’ sleep as dementia advanced. For a few, like Ruby, the person they were supporting slept long hours in the day, and so the severity of the situation was not regarded as so great at the time:

I can see he wasn’t sleeping terribly well. Excepting during the daytime… I’d hardly see him, you know, I’d have to wake him up to give him his lunch. And wake him up to give him his dinner. (Ruby, 86).

She went on to reflect:

as far as sleeping goes, the sleeping that I noticed the most was the sleeping all day and then still sleeping at night but not as soundly. (Ruby, 86).

Ruby associated this more with his mood and antisocial behaviour with “the rot” of dementia. And could maintain her sense of autonomy in the day. However, others, such as Nathalie, noticed a more disruptive decline in sleep and their own sleep being affected. Here Nathalie illustrated themes of timing of sleep being limited, her uncertainty of sleep as well as the progressive decline:

In those earlier days it wasn’t so bad. I wouldn’t know often if he’d been in and out and sometimes I did, and sometimes I didn’t and then he started, he would just…. He would go to bed really early. He’d be awake early, you know at 11 o’clock. He’d go to the toilet. He couldn’t go back to sleep. Or he’d get up and wander round. And then he might go back to bed. And then it just became progressively worse really…

So, what was happening was that he was, he wanted to sleep during the day cos of course he was tired. He’d get up in the morning and want to go back to bed after breakfast. So, you know, we had a real tussle about that. ‘Cos I knew that if he did that, he wouldn’t sleep. So, we did a bit of compromising and he could go back for an hour. But then I used to haul him out. But then in the afternoon he’d want to go back again. And so then what was happening at those times at night is that he was going to bed quite early. I would try and keep him up till at least 8 o’clock and that was really difficult. He used to get really querulous and, and cranky. And yeah and then he would – sometimes he might sleep for about four or five hours and be awake at 2 o’clock. But other times he might sleep for an hour and be awake for... And so, and then he’d get up and down, up and down, up and down. And then I – as things progressed, I'd find him in the shower. I'd find, he will have left the house. He’d be out in the shed looking for things that didn’t exist. He would be checking the house because he was hallucinating a lot. He thought that people were in the house. And so when it got to the stage of him getting up and down a lot, that’s when my sleep was really affected. (Nathalie, 64).

The theme “*I was tired but I had no choice*” illustrates how, even when carers were aware of the impact of care on their sleep, they simultaneously described the limited options for facilitating their own sleep or wellbeing. Many described functioning on “autopilot” or “high alert” with a strong will to prioritise and maintain the wellbeing of the care recipient at home for as long as possible:

I was like an autometer really. I was just working, looking after his needs. And thought I was caring about myself as well. There were days when I was very shaky. (Pamela, 75).

Regarding management of sleep problems, the theme *“I tried lots of things”* was used to describe strategies participants trialled to improve their own sleep or that of the person they were supporting. They were resourceful and pragmatic with their attempts to find solutions to monitor and/or ensure the safety of their family member in the night:

I needed some kind of alarm, which I built … these patterns developed as each stage what was happening, then you found a strategy to keep things going...what happens if I die in the middle of the night?... I needed to make sure Linda was protected. (John, 83).

Although strategies were important, they could not address all possible outcomes. Even when in-home care was available, support to improve sleep was seldom mentioned and the timing of assistance was not usually appropriate to enable sleep for the carer. Time in respite care was often limited with regards to enabling sleep recovery. Furthermore, as Pamela describes, sleep disturbances were often exacerbated during or after respite care, due to the upheaval and adjustment it entailed:

I had very little respite care because he wouldn’t want to go. And you know, that, people used to say, ‘he needs to go, you need to make him go’. But I mean, he would get aggressive and it wasn’t easy. I've got him into one rest home. They rang me at one o’clock in the morning and said ‘can you come up’. And I said well, ‘I wouldn’t have put him there’. And for two nights, because I need the sleep, if I had thought I was going to come up. But anyway, I had to go up. So that was a whole night’s sleep I didn’t have there from one o’clock through till five I think I left. Which is unfortunate. (Pamela, 75).

### Sleep around Transitions to RAC

A pivotal aspect of the interviews were discussions around the period when the person with dementia moved into RAC. Specific events and reasons for the transition were varied. Examples included realising additional support was required, to a more direct medical event which led to hospitalisation and reassessment (see Wright, 2020).

The theme *“Some teary nights”* reflects how, for many, this period was one of heightened stress, inability to relax and upset:

People used to say ‘oh you look really calm’ and everything. Oh my god, like the turmoil underneath was huge. And I've never, I've never been able to relax. So, of course, it just was, everything came out. It was heightened. My non-relaxation became even worse. But became very obvious as well…So the only avenue out for me was like residential care. (Nathalie, 64).

Nathalie points to the mismatch between her outward appearance of calm and the inner struggle to manage sleep alongside dementia caregiving. Others described complete exhaustion (within the theme *“I just crashed”)* contributing to their coming to terms with decisions around the move:

Oh, a horrible decision. And I've put it off as long as we could but. In the end I was just a zombie. (Daniel, 73).

Immediately after the person with dementia had moved into RAC, many carers described a continuation of this period of mental exhaustion either through a state of relief and catching up on sleep *(“sleeping like a log”),* or more of a debilitating effect of sleep deprivation taking over:

honestly, I was so tired I couldn’t put one foot in front of the other. (Pamela 75).

Others maintained a façade of managing the situation throughout, not wanting to admit to others how they had been affected.

I think that first month I just was absolutely shattered, and I didn’t want to admit that to family. (Eva, 24).

“*I should have prioritised my sleep”* was a common theme representing how participants recognised that they had been more sleep deprived during their active caregiving than they realised. It wasn’t until the time of the transition that they identified how much of an impact caregiving had had on their sleep and waking function:

I don't think I could have gone over, gone on much longer. I think I probably would have just collapsed and died. That’s how tired I felt. …And yet while he was here, and I was looking after him I didn’t feel it. (Pamela, 75).

Carers retrospectively acknowledged the impact that sleep deprivation had probably had on their health, mood, or wellbeing. Sleep debt was identified as ultimately affecting their ability to manage the overall caregiving situation. For example, Nathalie (64) originally thought her cut off point for care would be continence-related; in hindsight she felt sleep deprivation was crucial, whilst acknowledging that recognising the impact of sleep is hampered during active caring:

So you think you're doing all right. And sometimes I'd be out in the car and I think ‘I wonder if people out here know how dangerous I am’. You know. I've only slept two hours for the last, you know, two nights or something. And, and I mean I know that’s terrible and I shouldn’t have been out driving, but it was the only way that I could manage with him, get him in the car. Take him to the beach, give him an ice cream…

And that’s the other thing too cos of course you just adjust slightly each time as well. Okay cos initially I think ‘oh, god I've only had five hours sleep’. And I'd think that was terrible. And now, or then as time got on. If I got two hours in a row, I was like ‘yay’! You know, like, so, you do of course. And I guess you do that with children too. You have periods where you know, sleep, sleep isn’t, isn’t the main thrust really. Nathalie (64).

### Sleep Post Transition

After the transition, the context of sleep changed but it did not necessarily improve. The theme *“I kept busy”* represents how many carers reported using their newfound time for catching up domestically or making big life decisions. At times, sleep remained compromised due to the busy or stressful situation (for example through moving or renovating the home), with the assumption of *“It’s now or never” (Sarah, 82)*. Even once the person with dementia had transitioned to RAC, some carers continued to regularly visit or anxiously waited for news from the care facility.

I think part of me was waiting for the phone call. I'm guessing because I don’t know. But I found myself at times still sitting here in the chair. (John, 83).

They said [Staff at care facility], 'When things go wrong, he calls for you'. So, I guess that sort of – I'm getting no closure, and I guess that's why sometimes I sleep better than others…. So, he's not here but he is still here. (Sally, 77).

Many described the situation post transition as liminal with a common theme being that carers *“responsibilities don’t end”.* This was associated to feelings of grief, guilt, as well as concern for the person with dementia, which affected carers’ ability to get to sleep and stay asleep. Themes of missing the person with dementia, having concerns regarding their wellbeing, and getting used to no longer needing to sleep on such high alert were key. The grieving process also triggered solutions for overcoming sleep problems around this time:

What I resorted to do, is just putting a couple of pillows where she used to sleep and a bit of blanket over that, so I just felt as though there was another person there. (Daniel, 73).

Like all wives’, you’ve suddenly become widowed. And you do all the things that widows do. You sniff their clothes. You cuddle their pillow. Because you miss them. (Sonia, 68).

This theme reflects how sleep continued to be affected due to the lasting impact of the disrupted schedules and habits created through the caregiving situation. This made it challenging to fall asleep and stay asleep despite no longer being woken by their family member with dementia:

Do you know what I did notice? It took me a long time, I suppose a month, not to reach out to see if he was still in bed. I woke up because it was all so quiet, and I'd reach out and there was nothing there, and I'd sort of …. So, I sleep in the middle of the bed now and that stopped me…. Although he's in care, I'm still in the position of living on eggshells. (Sally, 77).

Carers adaption following their family member moving to RAC was not immediate, as Sally illustrates. The quietness was unnerving and disrupted her sleep and she adjusts to this by shifting position in the bed. Although this prevents her night waking, she experiences life with a partner in RAC as unsettling.

A key theme concerning sleep status post transition was that carers reported “*sleeping on my time”* which indicated some sense of improvement*.* The luxury of being able to reclaim a sense of control and identity around sleeping as well as waking life was expressed with regards to timing and routines. This included being able to comfortably lie awake at night if need be and the freedom to nap in the day:

Well, it's in my time, you know. And if I'm awake then, it, that’s fine because that’s my natural rhythm. And I'm used to that. And if I want to get up and have a cup of tea and a piece of cake at two in the morning, absolutely fine. You know, it's not a problem. (Sonia, 68).

Others, like Sarah, spoke of regaining natural rhythms of sleep, linking sleep post care to the descriptions of sleep before care began:

here I am...I'm on my own. I'm not disturbing anybody...I'm so grateful for all of this…This is a new acquisition this chair. And I thought I need to be able to have a somewhere where I can comfortably…put my feet up and just have a little nap in the afternoons. (Sarah, 82).

Here, the regaining of a sense of place and personal autonomy is illustrated with the purchase of a chair as a place to sit undisturbed and nap on her own terms.

With the increased opportunity to sleep, many reported an influx of dreaming. The theme *“I’m dreaming, dreaming, dreaming!”* encompasses the mixed experiences here*,* with some having higher dream recall and intensity, adding to the luxury of greater sleep opportunity and experiences:

I wake up in the morning and I'm dreaming and dreaming and dreaming. My mind's so busy! I wake up actually exhausted. (Sally, 77).

However, many described the dream content as unpleasant. Nightmares associated with the person with dementia, changed family relationships, and health were common:

…it was reoccurring, nightmares effectively, and it was about mum, you know, I'd imagine her dead or dying, like all the horrible stuff, or they wouldn't make sense and it would be like looking down and seeing her being hurt and I was up on a hill and couldn't get there. …it's weird because I don't normally remember my dreams. (Eva, 24).

Some reflected that their dreams perhaps were a representation of their waking feelings or a normal response to a challenging event:

I do have, which I didn’t have before…unpleasant dreams…I don’t know, I mean it could be that I’m feeling that I’m inadequate that I haven’t supported her properly. (Steven, 83).

Edward comes back and haunts me sometimes. …which is only natural I would think. (Grace, 69).

Finally, the theme *“my sleep affects my health”* represents how, post transition, carers typically acknowledged that they couldn’t’ have managed any longer usually due to their own health issues or decline:

I couldn't look after myself hardly, let alone her as well. (Olivia, 73).

Many indicated an acute awareness of the importance of sleep with regards to their own health, wellbeing, and safety. They also presented insights into the factors which could affect their sleep and knowledge of recommendations around sleep management:

Overall, I can only draw one conclusion, that's my conclusion of course, the whole way of living affects your sleep, affects your rest, and I realise now that there must have been times that, yeah, I was tired and didn't really function as well as I should. (Jacob, 87).

Invited to look back over his experience of caregiving, Jacob concludes that he struggled to function at times but was unaware at the time. Only by assessing sleep within the trajectory of caregiving did he identify these times of exhaustion.

Together these themes reflect the chronic impact that sleep disturbances have during active caregiving. There is a rebalancing of sleep to align with the original patterns and preferences of the carer. Some admitted that sleep disruptions remained which were related to worry or concern for the care recipient’s wellbeing:

I think I might sleep when she dies, you know, because it's all over then. (Olivia, 73).

### Sleep Post Death

For eight participants, the care recipient had died since moving to RAC. The events around death were diverse and personal. The theme “*I miss them dreadfully”* represents how bereavement was commonly described as affecting ability to get to sleep and stay asleep. This was typically presented as normal and a natural part of the experience of bereavement, which would likely resolve with time:

I've never lived on my own. And I've, I am finding it quite difficult being on my own. That’s what life’s about so… (Grace, 69).

I still haven't established a pattern… I suppose in time that will all settle down. It has to. (Daniel, 73).

The theme *“A sense of closure offering space for sleep”* reflects the other common comments concerning this time. Sleep was slowly improving with the adaptation to the nights and days without the high physical and mental responsibilities of caregiving described in the earlier themes, and a period of rebuilding the self and lifestyle was beginning:

Since Linda died about nine weeks ago, I’ve slowly been catching up on my sleep. I reckon I’ve got four years of sleep to catch up on. Getting to the stage now where I’m waking up in the morning and actually feel like getting up. I thought “I’ve had enough sleep”, you know, “I can get up now”. (John, 83).

John describes his waking pattern after care as being able to decide that he has had enough sleep. This illustrates the shift from sleep as dependent on his wife’s needs and priorities to re-establishing his own needs for rest. These themes indicate the long-lasting impact of the responsibility of caregiving and associated grief beyond the active period of caring in the home.

## Discussion

This study explored the changing sleep status of those caring for a family member with dementia. At time of interview, when opportunities to sleep were beginning to recover, carers still reported issues. Indeed, pre-interview surveys indicated that the majority (85%) scored above the PSQI threshold indicative of ‘disturbed sleep’. Surveys of the general older New Zealand population indicate prevalence of self-reported sleep problems in the range of 20%–40%, depending on the scale and population (Gibson et al., 2015; Gibson, Gander, et al., 2020).

Rather than taking a cross-sectional measure of sleep status, or looking a single point in time, the focus of these analyses was understanding sleep across the trajectory of care using retrospective interviews. This foregrounded the history and transitional nature of both caregiving and sleep. The findings demonstrate that the nature of sleep disturbances, their effects, as well as their self-management change with the progression of time and dementia. These analyses illustrate how the self-sacrificing nature of the carer role extends to their management of sleep. The balance of sleeping and waking life and associated personal preferences was compromised in favour of supporting the person with dementia as well as possible. While caring, sleep was represented as a luxury often forgone to achieve what was required in waking life. Yet, retrospectively, sleep was identified as a key factor affecting the overall situation. From this vantage point, carers recognised exhaustion and attempted to meet their needs for sleep and regain their natural sleeping patterns.

When actively involved in supporting a family member with dementia, carers’ sleep was typically described as becoming progressively disrupted. Due to the unpredictable nature of dementia-symptoms routines and the continuous responsibilities, caring can cause a state of high alert despite sleep deprivation. Many carers attempted to facilitate better sleep and wellbeing for those with dementia, often compromising their own self-care. Retrospectively, carers reported being exhausted. At the time of transition, carers either reported a period of crashing, or the continuation of being or feeling busy. Grief was common throughout. After the active caregiving role was relieved symptoms of insomnia, poor sleep habits, and nightmares were common. Yet many were optimistic that sleep would recover with the grieving process and were enjoying the luxury of achieving sleep within their own timing and preferences. An influx of dreaming or nightmares was common after the person they had been caring for moved into RAC. This may be explained by either an intense physiological resurgence of rapid eye movement sleep after a period of significant sleep deprivation, and/or the impact of intense emotions in waking life being reflected in during dream experiences which, in turn may act as a form of positive cognitive processing and recovery of grief (Scarpelli et al., 2019).

The themes constructed here expand on sleep issues previously identified during dementia care (Aber & Venn, 2011; Gibson et al., 2014; Gibson & Gander, 2020; Leggett et al., 2018; McCurry et al., 2015). For example, in a large cross-sectional study, Okuda et al (2019) found that dementia-related symptoms such as night awakenings, snoring, and daytime sleepiness negatively impacted carers waking and sleeping wellbeing. Through using retrospective interviews, the present study deepens our understanding of the subtle changes to sleep and wellbeing throughout care and highlights the long-term effect of caregiving on sleep status, beyond the transitions to formal care or even death. Therefore, this study offers unique insight into the psychosocial relationship between symptoms of dementia, caregiving, and sleep status and their interplay with the innate physiological need for sleep and its importance as a pillar of health.

These findings reflect the nuanced individual differences in circadian status and sleep preferences alongside the importance of autonomy in managing time, activities and sleep. Previous research has identified the impact that emotional caregiving difficulties, role overload, and symptoms of depression have on heightened sleep disruptions among informal dementia carers which can exist regardless of whether they live with the care recipient (Simpson & Carter, 2015) as well as beyond the caregiving role (Leggett et al., 2018; Von Känel et al., 2012). For example, in a survey of American former dementia carers, Corey et al (2020) revealed the long-lasting impacts of self-identified dysfunctional coping on sleep and mood. Follow-up interviews in a sub-sample resulted in a key theme around ‘learning to live again’ whilst managing the long-lasting impacts that dementia-related caregiving had had on sleep and health (Corey & McCurry, 2018).

The present findings also provide empirical examples in support of sociological concepts of sleep and sleeping. For example, understanding sleep time as inactive and unproductive as opposed to active or productive wake time can be located within the expectations of an increasingly busy modern society (Hsu, 2014; Williams, 2007). With ageing and reductions of paid work, the pressure for productivity is socially assumed to decrease. Yet for informal carers, many of whom are older, periods of rest and sleep need to be balanced alongside guardianship responsibilities as well as their own health and wellbeing. Similarly, the findings speak to theories around negotiating sleep within the individual, dyadic, familial, and wider social contexts (Meadows, 2005). Here, the mediating context of dementia care shapes carers agency for their own sleep practice, highlighting how those we live and sleep with can support or challenge sleep needs and preferences. For example, carer’s reports of their inability, or lack of desire to rest during the daytime represented here support notions of daytime sleep as negative connotated with age-related decline, laziness or inability to cope with waking responsibilities (Venn & Arber, 2011). The interviews and corresponding transcripts were rich with stories that were outside of the scope of the present thematic analysis. A separate narrative analysis was conducted exploring the overarching tensions of balancing individual sleep needs and preferences with the vulnerabilities of falling asleep potentially undermining the responsibilities of guardianship (see Gibson et al., 2022).

### Considerations

The present findings reflect remembrances after the care recipients had moved into RAC, and for some participants, after the care recipient had died. It is important to consider how the stories told are shaped by knowledge, memory, understandings and meanings which shift over time. For example, emotions are likely to be described in a less intense manner compared to when stories of recent intense events are elicited (Breheny, Horrell & Stephens, 2020; Horrell, Breheny & Stephens, 2020). The advantage of retrospective interviews was in gathering the sleep story after the transition to formal care, putting each stage into perspective. This is particularly powerful in the context of dementia caregiving as significant sleep deprivation impairs our ability to recognise the effect of sleep on our waking function, which has implications for fatigue-related incidents (Boardman et al., 2018). Carers here noted that, in hindsight, their sleep should have had greater priority, indicating that if asked about sleep in the midst of care responsibilities, they may have been unlikely to recognise their sleep deficit in the same way. Together these factors are important when considering carers claims that they are coping while caring.

These findings also help us understand the importance of consultation and timely introduction of sleep-related support and interventions. Previous research indicates that non-pharmacological interventions show promise, for example addressing dementia-related sleep disorders and sleep disturbances secondary to caregiving using timed bright light therapy, exercise and/or sleep education (Gibson et al., 2017; Song et al., 2021). However, such interventions are typically more feasible and successful when a dyadic approach is taken at a time when the family believe that sleep health is an area that they need to address as well as accommodate changes to routines or sleep practices.

The themes constructed here, while pertinent to the dementia care situation, will not apply to all carers. Shared characteristics of the participants may have shaped the comments. Strategically recruiting some (35%) rural-dwelling carers within this research provides some diversity of experiences. However, all recruitment took place through opting into a mailing list from a survey originally distributed via regional dementia organisations (Gibson & Gander, 2020). Families with limited access to diagnostic services (for geographical or financial reasons), of Māori or Pacific Island ethnicity, with poorer physical and mental health, and those who do consider the ‘dementia’ and ‘carer’ terminology differently are less likely to engage with dementia-related organisations and services (Dudley et al., 2019; Dyall, 2014). They are also less likely to engage with research activities (Beattie et al., 2018). These carers require greater attention for future dementia-related research to explore experiences and needs of those who are not connected to regional service providers. Their disconnection from service provision may mean they experience different sleep disruptions and needs.

In the present study, one carer identified as Māori and one as Samoan. They differed from the remaining sample in that they were younger and supporting a grandparent or parent who they had moved in with rather than being in a long-term co-residing, spousal relationship. While the impacts on sleep are likely to be similar with regards to dementia symptoms and management, nuanced cultural differences have been identified in beliefs and family practices around ageing and dementia care as well as access to services (Dudley et al., 2019). Māori have been identified as more likely to experience sleep disorders as well as earlier onset of dementia-related disease compared to NZ Europeans. Yet Māori are less likely to have a formal diagnosis or access support services (Dyall, 2014; Paine, Gander, Harris & Reid, 2004, 2005). This has been related to differing perceptions of sleep, ageing, and dementia but also living in a society which is less responsive to Māori needs (Dudley et al., 2019). With most of the present sample being NZ European, the themes described do not necessarily reflect the diversity and severity of sleep issues among families affected by dementia in NZ. Research considering the balance of sleep and wake within different cultures, for example within intergenerational and whānau (extended family) based care, is of interest in order to represent the reality of dementia care in Aotearoa NZ.

### Implications

This research illustrates the changing sleep status of families affected by dementia as well as the effects of sleep disruptions and strategies to support sleep and wellbeing. Recommendations from NZ and elsewhere concern a common goal of “living well with dementia” within the family home for as long as it is feasible and safe (Martyr et al., 2018; Ministry of Health., 2013). Descriptions of sleep throughout the trajectory of care demonstrate the importance of sleep as a factor for living well, yet also something that is frequently compromised, disrupted, and difficult to manage due to the complexities of the situation. Many described themselves as so overstimulated and out of routine by the time the transition to RAC occurred that they struggled to sleep regardless of the opportunity. Findings from this study may help form advice to increase recognition of the importance of sleeplessness and fatigue. This could include prioritising lost sleep from an early stage, through strategic planning for sleep and external support which facilitates sleep.

The themes outlined here highlight the nuances and complexities of sleep within informal dementia care. This research gives voice to the caregivers, particularly around the complex time of their family member moving into RAC as well as their experiences thereafter, which are seldom considered. These findings add to growing research incorporating sleep into frameworks of dementia care and considering both those with dementia and their family carers. Findings will be used to inform person-centred, timely and appropriate approaches to support families affect by dementia.
